# Supplementary material for: Participatory science methods to monitor water quality and ground truth remote sensing of the Chesapeake Bay
Source: PLoS One. 2024 Oct 31;19(10):e0305505. doi: 10.1371/journal.pone.0305505 (PMC11527148; doi:10.1371/journal.pone.0305505)
Supplement: S2 Fig — (PDF) [file pone.0305505.s005.pdf]

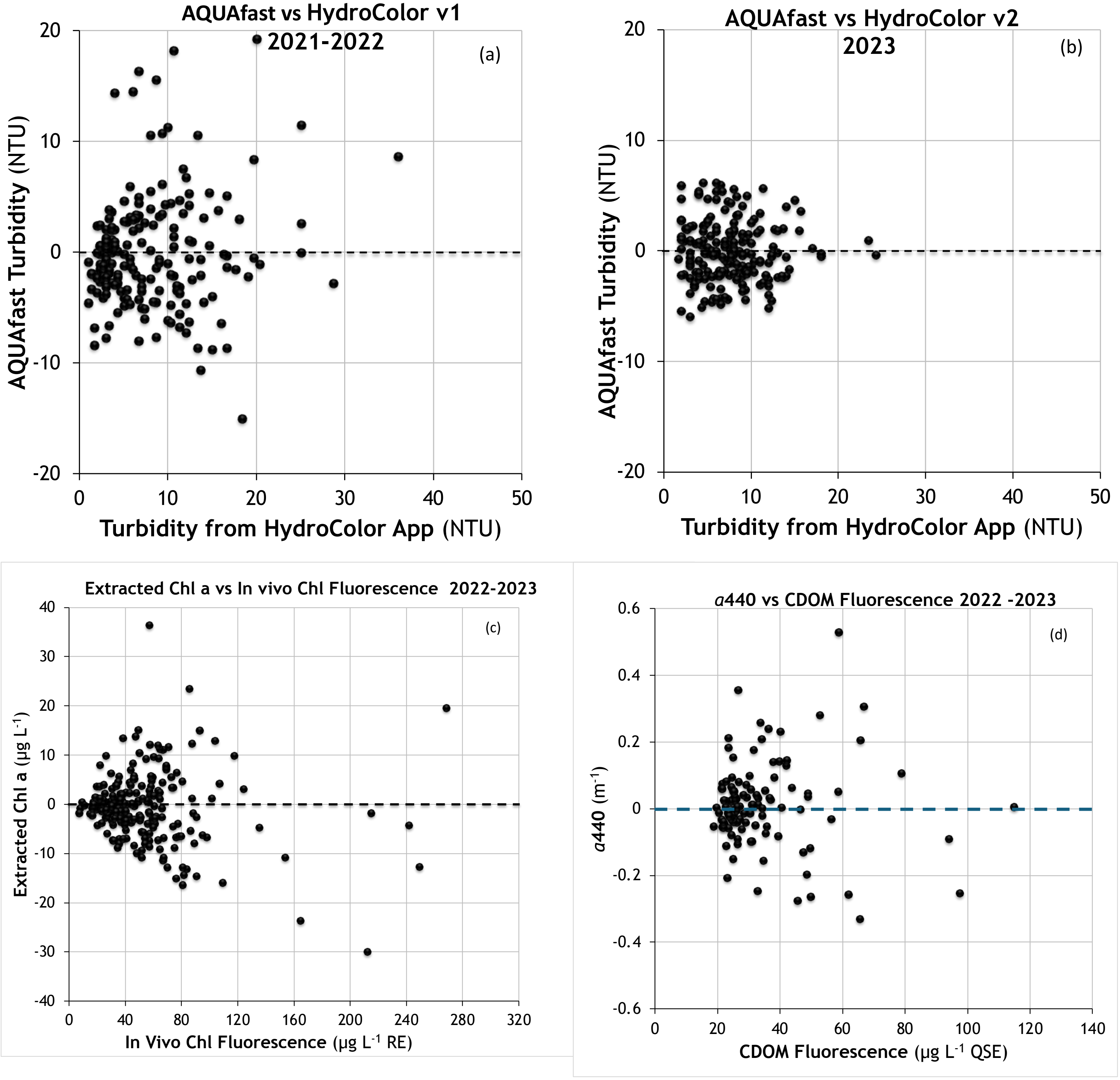

S2 Fig. Plots of the residuals for the regressions of validation vs volunteer measurements, based on equations listed in Table 2, (a) AQUAfast (NTU) vs HydroColor v1 (NTU), (b) AQUAfast (NTU) vs HydroColor v2 (NTU), (c) Extracted Chl a (µg L-1) vs Aquafluor in vivo Chl fluorescence (µg L-1 RE), (d) CDOM absorbance at 440 nm (a440) vs Aquafluor CDOM fluorescence (µg L-1 QSE).
